# Recent advancements in integer-valued autoregressive models for count data time series: A comprehensive review

**DOI:** 10.1016/j.mex.2026.103805

**Published:** 2026-01-26

**Authors:** Vinitha Serrao, Satyanarayana Poojari, Asha Kamath

**Affiliations:** Department of Applied Statistics and Data Science, Prasanna School of Public Health, Manipal Academy of Higher Education, Manipal, Karnataka, India

**Keywords:** Count data time series, Underdispersion, Overdispersion, Zero-inflation, INAR (1) model

## Abstract

Count data time series, characterized by non-negative integer values, frequently arise across diverse domains, including finance, public health, economics, epidemiology, and environmental sciences. Such series often exhibit characteristics such as equidispersion, overdispersion, underdispersion, and zero-inflation/deflation. Failure to appropriately account for these features can result in biased parameter estimates and misleading statistical inference. This review presents a comprehensive overview of recent methodological developments in integer-valued autoregressive (INAR) models, with particular emphasis on thinning operators, estimation methods, and model extensions. A systematic literature search was conducted using electronic databases, including Scopus and Google Scholar, to identify relevant studies published between 2010 and 2024. Recent research has primarily focused on the development of novel thinning operators and flexible innovation distributions aimed at constructing unified modeling frameworks capable of accommodating multiple characteristics of count data simultaneously. This review highlights prevailing research trends, identifies existing methodological gaps, and outlines promising directions for future research in count data time series modeling.

## Graphical abstract

**Figure:**
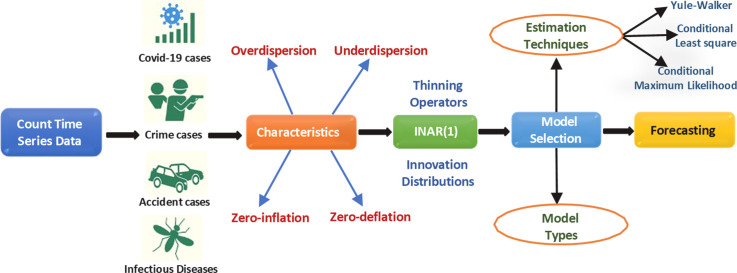

Specifications table.

**Table:** 

**Subject area**	Mathematics and Statistics
**More specific subject area**	Time Series Analysis
**Name of the reviewed methodology**	Models addressing various dispersion patterns, zero-inflation, and zero- deflation in count data time series.
**Keywords**	Count data time series, Underdispersion, Overdispersion, Zero-inflation, INAR (1) model.
**Resource availability**	Not Applicable.
**Review question**	• What are the existing modeling approaches for handling various dispersion patterns in count time series data, and how do these models differ in terms of structure, innovation distributions, thinning mechanisms, and estimation methods? • What are the existing modeling approaches for handling zero-inflation and deflation patterns in count time series data, and how do these models differ in terms of structure, innovation distributions, thinning mechanisms, and estimation methods? • What are the key developments in thinning operators used in INAR (1) models?

## Background

Count data time series, consisting of discrete events recorded at regular intervals, are prevalent across many scientific fields, including road accidents [1], economic indicators [2], COVID-19 deaths [3], influenza cases [4], and criminal incidents [5].These applications are closely linked to several SDGs, including SDG 1 (No Poverty), SDG 2 (Zero Hunger), SDG 3 (Good Health and Well-Being), SDG 8 (Decent Work and Economic Growth), SDG 11 (Sustainable Cities and Communities), and SDG 16 (Peace, Justice, and Strong Institutions). For example, analyzing road accidents can inform strategies to improve urban safety and transportation planning, monitoring disease cases can support public health initiatives, studying crime patterns can strengthen justice systems and examining economic indicators can identify vulnerable populations and guide poverty alleviation programs. By providing insights into these critical areas, INAR models can help guide evidence-based policies that contribute to achieving the SDGs.

**Fig. 1:**
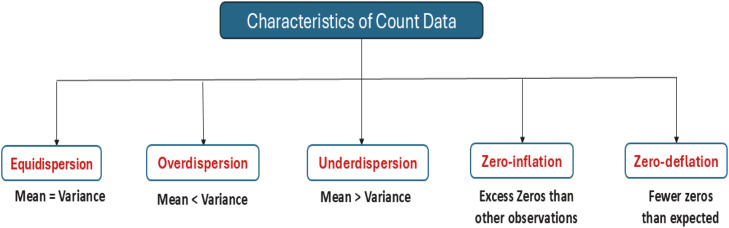
Characteristics of count data.

One of the main challenges in modeling and forecasting count time series is addressing unique features such as equidispersion, overdispersion, underdispersion, zero-inflation, and zero-deflation, as clearly illustrated in Fig. 1. If these features are not properly addressed, the resulting forecasts may be inaccurate, potentially leading to misleading conclusions [6].

For large count datasets, continuous time series models like ARIMA can be applied, leveraging the central limit theorem, which states that discrete distributions (e.g., Poisson, Binomial, Negative Binomial) converge to a normal distribution under certain conditions [7]. However, ARIMA models are often unsuitable for count data and discrete-valued series because they assume real numbers and normally distributed errors. As a result, specialized models for count data have been proposed, although they still face limitations in addressing overdispersion effectively.

To address these limitations, INAR models introduced by McKenzie [8] and Al-Osh and Alzaid [9] have gained prominence. These models extend classical autoregressive models by incorporating integer-valued distributions, which enable them to handle serial correlation and capture the distributional properties of discrete data more effectively. The INAR (1) model is represented as:

$$X_{t}=\alpha \circ X_{t-1}+\varepsilon_{t};\;0\leq \alpha <1$$

where $X_{t}\;$ is the observation at the time t and the operator (∘) is referred to as the binomial thinning operator, given by [10]. This operator indicates that there is a probability α∈[0,1) of a previous count being carried over to the current period. This relationship can also be expressed as $\alpha \circ X_{t-1}∼B\left(X_{t-1},\alpha \right)$ where $B(X_{t-1},\alpha )$ represents a binomial distribution with $X_{t-1\;}$ trials and success of probability α. Additionally, $\left\{\varepsilon_{t};t\in Z\right\}$ represents a sequence of uncorrelated, non-negative, integer-valued random variables characterized by a mean of $\;\mu_{\varepsilon }$ and a variance of $\sigma_{\varepsilon }^{2}.$

To illustrate parameter estimation in the INAR (1) framework, consider a public health application in which $X_{t}\;$ denotes the daily number of influenza cases in a region. One commonly used estimation approach is CLS, based on the conditional expectation

$$E\left(X_{t}|X_{t-1}\right)=\alpha X_{t-1}+\mu_{\varepsilon }.$$

The CLS estimators of α and $\mu_{\varepsilon \;}$ are obtained by minimizing the sum of squared deviations between the observed counts $X_{t}\;$ and their conditional expectations. For instance, an estimated thinning parameter $\hat{\alpha }=0.65\;$ implies that, under the binomial thinning operator, approximately 65% of the cases from the previous time point are expected to persist into the current period. The estimated innovation mean ${\hat{\mu }}_{\varepsilon }$reflects the average number of new cases arising independently of past observations.

YW estimation provides an alternative moment-based approach, exploiting the theoretical relationships between the mean, variance, and autocovariance of the INAR (1) process. Due to its computational simplicity, the YW method is particularly suitable for large datasets. When the distribution of the innovation process $\varepsilon_{t}\;$ is specified, CML estimation can also be employed and generally yields more efficient parameter estimates.Although the INAR (1) model is considered efficient, it still faces challenges, particularly in addressing overdispersion, underdispersion, and zero-inflation, which are common in real-world count time series data. Traditional count data models often struggle with these complexities, resulting in inadequate model fit and poor predictive performance. This review explores advancements in INAR (1) models from 2010 to 2024, specifically those designed to address equidispersion, overdispersion, underdispersion, and zero-inflation/deflation in count time series. By examining a broad set of characteristics within a single framework, the review helps researchers identify suitable models and estimation techniques aligned with specific data characteristics, thereby improving estimation and forecasting accuracy. This comprehensive review offers a clear understanding of the strengths and limitations of existing models and provides a foundation for identifying research gaps and guiding future developments in count time series modeling.

## Method details

### Search strategy

The articles reviewed in this manuscript were obtained from the Google Scholar web search engine and the Scopus database, covering the period from 2010 to December 2024.The search utilized keywords such as

i. "count data time series" AND "overdispersion"
ii. "time series of count" AND "zero-inflation" OR "zero-deflation"
iii. "count data time series" AND "overdispersion" AND "underdispersion".

Before the screening process, duplicate articles were removed to ensure a unique set of sources. The review focused on peer-reviewed journal articles, review papers, conference proceedings, and other key contributions published in the English language. This process resulted in a total of 326 articles, including 299 articles from Google Scholar and 27 articles from Scopus.

The screening process was conducted in stages, beginning with an evaluation of the article titles, followed by the abstracts, and finally the methods sections. This process resulted in a final set of 42 articles selected for detailed review. The specific inclusion and exclusion criteria are outlined below.

## Inclusion criteria

• The title and abstract include relevant keywords
• The article is written in the English language
• The article is published in a journal or conference proceedings
• The study includes the development of relevant methods

## Exclusion criteria

• Duplicate articles
• Articles written in languages other than English
• Articles addressing unrelated methods
• Articles that are not accessible

**Fig. 2:**
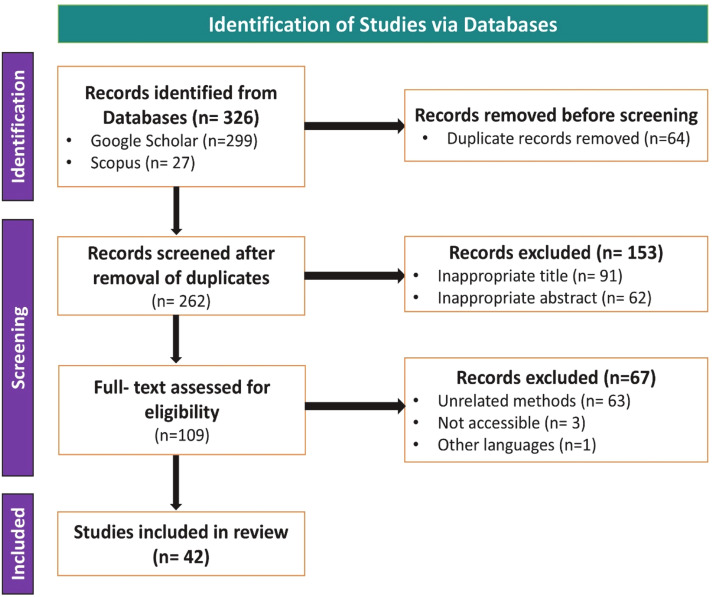
PRISMA flow chart.

A total of 326 articles were initially retrieved, and after applying the inclusion and exclusion criteria, 42 articles were selected for this review. The procedure followed during the article selection process is presented in Fig. 2.

**Question-1:** What are the existing modeling approaches for handling various dispersion patterns in count time series data, and how do these models differ in terms of structure, innovation distributions, thinning mechanisms, and estimation methods?

## Models addressing various dispersion patterns

This section is organized into four categories: (1) models specifically suited for handling overdispersion; (2) models designed to address underdispersion; (3) models capable of managing both overdispersion and underdispersion; and (4) models that simultaneously account for equidispersion, overdispersion, and underdispersion. This structured approach provides researchers with a clear framework to select the most appropriate models and estimation methods based on the distinct characteristics of the count data time series they are analysing. By categorizing models in this manner, it enhances the ability to choose suitable techniques for more accurate analysis and forecasting.

### Models handling overdispersion

Overdispersion is a common issue in medical studies, often resulting from positive correlations between monitored events [11]. To handle overdispersion, various statistical models have been developed. The Negative Binomial distribution is commonly employed for modeling count data that exhibits overdispersion followed by quasi-poisson, and zero-inflated models ( [[12], [13], [14]]). However, these models sometimes underestimate standard errors and overstate the significance of certain covariates. To address the challenges of modelling overdispersed count time series, INAR models have become increasingly popular. To account for overdispersion, two main approaches have been proposed:

a. modifying INAR (1) models by using alternative marginal distributions and thinning operators
b. adapting the binomial thinning operator to handle different innovation distributions.

Various INAR (1) models address overdispersion in count time series. Initially, [15] proposed the NG-INAR (1) model, which uses a Negative Binomial thinning operator (∘) to extend the PINAR (1) model. Key estimation methods include MLE, YW, and CLS. The INAR (1) model with Geometric innovations proposed by [16] adopts CML and AFML methods for parameter estimation. By introducing a generalized binomial thinning operator ($\circ_{\theta }$), [17] formulated the first-order dependent counting geometric INAR model. The study employed CLS and MLE methods for parameter estimation. As a further advancement, [18] introduced a CP-INAR (1) model with a binomial thinning operator (∘). A test for overdispersion in INAR models was developed by using the property that a CP-INAR (1) model exhibits strong mixing properties characterized by weights that decrease exponentially. The INAR (1) model with a Poisson-Geometric marginal distribution, presented by [19] utilizes a two-step CLS estimation procedure to extend the functionalities of the PINAR (1) and GINAR (1) models. Building on this, [20] introduced a stationary INAR (1) model with Poisson-Lindley innovations, employing CLS, YW, and MLE for estimation. To accommodate heavy tailed count time series, [21] introduced an INAR (1) model incorporating Generalized Poisson inverse Gaussian innovations into the INAR(1) framework. This model accommodates both equidispersion and overdispersion and is also well-suited for count data with a high frequency of zero values with CML method for parameter estimation. Recently, [22] evaluated the performance of the ACP and PAR models, concluding that the ACP model is preferable across different sample sizes.

### Models handling underdispersion

Underdispersion can occur due to model overfitting or small sample sizes [23]. Several widely recognized parametric models are available for analyzing underdispersed count data. These include the Generalized Poisson distribution introduced by [24], the Double Poisson distribution proposed by [25], and the Conway-Maxwell Poisson distribution developed by [26]. Each of these models is characterized by two parameters. However, these models have limitations in effectively describing underdispersion. To address this issue, [27] explored the performance of a stationary INAR (1) process with two-parameter Good and Power Law distribution innovations, which demonstrated better properties for modeling underdispersed counts. However, extending these models to higher-order autoregressive structures, such as INAR(p) developed by [28], introduces further complexity. Notably, even if the innovations are underdispersed, the resulting INAR(p) process does not necessarily retain this property in the observed data. This limitation highlights an important direction for future research. Although underdispersion has received little attention in practical data analysis, it highlights the need for specialized models to effectively address this phenomenon.

### Models handling both overdispersion and underdispersion

Several models have been developed to address overdispersion and underdispersion simultaneously in count data, [29] introduced an INAR (1) model with power series innovations, using YW, CLS, and MLE for estimation. To address underdispersion within a hidden Markov framework, the Poisson- hidden Markov model was modified by [30] by replacing the Poisson distribution with the CMP distribution, resulting in the CMP-HMM model. A novel thinning operator based on the GSC distribution (⋄) was introduced by [31], leading to the development of an INAR (1) model estimated using CLS, weighted CLS, and MQL methods. In a related study, [32] introduced an extended binomial thinning operator (⊚), which featured two parameters, offering greater flexibility in modeling. Their work presented the EBINAR (1) model and explored two-step CLS estimation for the innovation-free case, alongside a discussion of CML estimation method. Later, [33] developed a flexible INGARCH model using the generalized CMP distribution which introduces an additional parameter to manage heavy tail behaviour, with parameters estimated using the CML method. Recently, [34] introduced an ZDGG-INAR (1) model. This model addresses the limitations of the GPIG-INAR (1) model and effectively captures all the characteristics such as zero-inflation/deflation, zero truncation, along with dispersion. The parameters of the ZDGG-INAR (1) model were estimated using methods such as YW, CLS, CML, and Bayesian approaches, making it a versatile tool for handling various count data characteristics within the INAR framework. This is the only model found in the literature that is suitable for all the characteristics of count data, and it is based on the INAR (1) framework.

### Models handling equidispersion, underdispersion and overdispersion

Models that address all three types of dispersion offer considerable flexibility and wide applicability in analysing various types of count data. The adaptability of the CMP model was emphasized by [35] using MLE for estimation. This model can also effectively manage both zero-inflation and zero-deflation in count data. Later, [36] developed an INAR (1) framework that integrates Bernoulli-Geometric marginal distributions and utilizes a generalized thinning operator ($\circ_{\theta }$), providing enhanced flexibility for count data modeling, with parameters estimated via MLE. Similarly, [37] proposed an INAR (1) model using Katz family innovations, which incorporates Poisson, Negative Binomial, and binomial distributions to capture a variety of dispersion patterns. Although both CML and MLE methods can be applied, the practical implementation of these models is challenging due to the complexity of the marginal distribution, which often lacks a closed-form expression.

Motivated by the ability of the Double Poisson and Generalized Poisson distributions to capture various dispersion patterns and their relatively simple probability mass functions, [38] expanded the INAR (1) process by developing the DP-INAR (1) and GP-INAR (1) models. This model uses Poisson innovations and binomial thinning (∘) to capture different dispersion patterns. Model parameters are estimated using methods such as CML, CLS, and YW, and a test is developed to identify the types of dispersion. A related extension incorporating a generalized Poisson thinning operator (⊛) was explored by [39]. Model parameters were estimated using CLS, MQL and CML methods. Additionally, [40] proposed a shifted INAR (1) model with Borel innovations. This model utilized binomial thinning (∘), with parameters estimated via YW, CLS, and CML methods and constructed a test for dispersion. More Recently the WNBL distribution based INAR(1) process presented by [5] combines binomial thinning operator (∘) with WNBL innovation, where the innovation distribution is constructed as a weighted combination of the traditional negative binomial and size-based negative binomial distributions, offering flexibility through adjustable parameters. Model parameters were estimated using CML, YW, and CLS methods and applied to real-world datasets from criminology and epidemiology, demonstrating its practical relevance and versatility.

**Question-2:** What are the existing modeling approaches for handling zero-inflation and deflation patterns in count time series data, and how do these models differ in terms of structure, innovation distributions, thinning mechanisms, and estimation methods?

## Models handling zero-inflation and zero-deflation

Count data frequently show an excess of zeros, known as zero-inflation, a concept formally introduced by [41] and [42]. Zero-inflated models, which combine a degenerate distribution at mass zero with a count distribution such as Poisson or Negative Binomial, were developed to address this issue. To address the problem of zero-inflation, [43] made significant advancements with the introduction of a generalized linear model using the ZIP distribution, which was specifically designed for a manufacturing process where additional zeros resulted from transitions between flawless and defective production states. Further, [44] expanded on this concept to include zero-deflation, thereby creating the zero-modified Poisson regression model.

The ZIP model is appropriate for datasets displaying equidispersion. However, for datasets with a high prevalence of zero counts, other distributions like the ZINB distribution [45] and the zero-inflated CMP distribution [46] have been developed as alternatives. The ZINB model addresses both excess zeros and overdispersion by considering population heterogeneity, where the probability of zero counts varies among individuals. Unlike the ZIP model, which is designed primarily for equidispersed data, the ZINB model is more suitable for datasets with both excess zeros and overdispersion [43].

Recent advancements include the development of various INAR processes to model zero-inflated and zero-deflated count time series. The- ZIP-INAR (1) model introduced by [47] merges the INAR (1) process with ZIP innovations to manage excess zeros using CML and AFML estimation methods. Related extensions within the INGARCH models were presented by [48], where ZIP and negative binomial INGARCH models were proposed and fitted to real-world datasets using the EM algorithm.

Within the state-space framework, [49] introduced a flexible state-space model that uses the ZINB distribution. Due to the limitations of traditional estimation techniques like the Kalman filter and Kalman smoother, the study utilized a Monte Carlo-based Expectation-Maximization algorithm, which includes particle filtering and smoothing for parameter estimation. The study also developed an R package ZIM (Zero Inflated Models) to support the application of this model. A new ZIP-INAR (1) model was considered by [50], where the PINAR (1) process was combined with an independent Bernoulli process utilizing YW, CLS, and MLE estimation methods. To address the shortcomings of the ZIP-INAR (1) model, [51] ZMG-INAR (1) model and introduced a test for detecting zero-inflation/deflation based on the model.

The innovation structure of the ZMG-INAR (1) model introduced by [51] is complex due to its intricate innovation structure and restrictive parameter conditions. To address this, [52] proposed an INAR (1) process with ZMG innovations, employing binomial thinning (∘). This model also accommodates equidispersion, underdispersion, and overdispersion, and its flexibility is enhanced by encompassing the GINAR (1) model as a special case. The parameter estimation used were CML, YW, and CLS. Additionally, flexibility in handling diverse zero patterns was achieved by [53] through the extension of the binomial autoregressive model - introducing four variants: (i) BAR (1) Process with zeros at random; (ii) BAR (1) Process with innovational zeros; (iii) Zero inflated binomial thinning for AR (1) model; (iv) BAR (1) Process with zero threshold. These models address various zero structures in count data using maximum likelihood estimation. Additionally, [54] proposed the generalized zero-modified geometric distribution along with a new thinning operator based on zero-modified geometric counting series, using maximum likelihood estimation. Finally, an INAR based model incorporating a randomized binomial thinning operator (∘) and a zero-inflated geometric distribution featuring random coefficients was examined by [55]. The model employs CLS and MLE techniques, providing greater adaptability for analyzing count time series with complex patterns.

Count data with an abundance of zeros and ones, common in sectors like insurance and industry, is relevant to SDGs 1 (No poverty), 8 (Decent work and economic growth), 9 (Industry, innovation and infrastructure), and 12 (Responsible consumption and production). Traditional INAR (1) models, including Geometric and Poisson variants, often struggle to accurately capture these data patterns. For instance, GINAR (1) models tend to overestimate zero counts and underestimate one counts, whereas PINAR (1) models exhibit the opposite behaviour. Additionally, existing zero-inflated and CP-INAR (1) models face similar limitations. To address these challenges, [7] introduced a novel mixture of GINAR (1) processes designed for overdispersed count time series with high proportions of zeros and ones. The estimation methods used were YW and QMLE. To further accommodate the higher prevalence of zeros and ones in count data, the zero-and-one inflated Poisson distribution was also introduced with the foundational contributions provided by [56] along with [57].Building on this, [58] proposed the ZOIP-INAR (1) model, which combines the ZOIP distribution with the INAR (1) process. This model effectively captures count time series with excess zeros and ones, as well as overdispersion or underdispersion.

A copula-based time series regression model incorporating covariates was introduced by [59] to effectively manage zero-inflation. Flexibility in modelling dependent count time series was enhanced by [60] through the development of a generalized Negative Binomial thinning operator (${*}_{\theta }$) with the parameter estimation conducted using YW, CML, and modified CLS methods. Expanding on the modelling of zero-inflated count data, [61] constructed an INAR (1) process with zero-modified Poisson-Lindley innovations, employing binomial thinning for zero-modified count time series, with estimation methods including YW, CML, and CLS. Building on this, [4] proposed a more comprehensive framework using zero-modified Power Series distributions within a Generalized Autoregressive Moving Average model. This approach provides flexibility, with parameter estimation performed using Bayesian methods via a Hamiltonian Monte Carlo simulation algorithm.

Additionally, [62] introduced a first-ordered integer-valued moving average process using Zero-modified Geometric innovations based on a binomial thinning operator (∘) for analysing count data with short-run autocorrelation. This model also effectively captures data exhibiting equidispersion, overdispersion, and underdispersion. Parameter estimation was performed using CML estimation method. A novel approach for modeling zero-modified count time series was introduced by [63] through the zero-modified stochastic conditional duration models,where latent Markov sequences generate intensities for zero-modified Poisson and zero-modified negative binomial distributions within a generalized state space framework for modeling count data with excess or deficit of zeros. Parameter estimation is facilitated through a generalized state-space form. Addressing the limitations of conventional INAR (1) models in modeling periodic count data, [64] introduced PZIP-INAR (1) model. This model specifically targets overdispersion caused by excessive zeros in time series with periodic patterns. By incorporating periodically varying zero probabilities within a Poisson process framework, the PZIP-INAR (1) model offers a more suitable approach for analysing such data, utilizing CML, YW, and CLS estimation methods. Finally, dependence in the thinning mechanism was proposed by [65] through- Neyman type A thinning operator (${⊛}_{\theta }$), resulting in a stationary INAR (1) process with a Geometric INAR (1) innovation structure, where parameters were estimated using CMLE, modified CLS, and YW approaches.

Recent advancements in integer-valued autoregressive models have improved the flexibility and accuracy of count data time series analysis, addressing equidispersion, overdispersion, underdispersion, and zero-inflation. The most commonly used estimation methods are CML, YW, and CLS. As complex count datasets continue to emerge, further development in model structure and estimation techniques will be crucial for capturing unique data characteristics and enhancing decision-making across various fields.

**Question-3:** What are the key developments in thinning operators used in INAR (1) models?

## Thinning operators

A widely used class of models for count time series is the INAR (1) family, which utilizes thinning operators. A thinning operator is a probabilistic tool that reduces a random count variable to a smaller integer-valued variable. This mechanism is especially effective for defining $X_{t}$ in terms of $X_{t-1}$ ensuring that $X_{t}$ retains its integer nature.

Table 1 provides an overview of various thinning operators, along with their associated mathematical formulations and corresponding practical examples, highlighting application scenarios in which these operators can be effectively employed. This facilitates a clearer understanding of their practical relevance. The table also illustrates the progression from binomial thinning to the GNTA thinning operator through the incorporation of increasingly flexible random variable distributions.

**Table 1:** Overview of thinning operators and their mathematical operations.

**Name along with proposed author**	**Operator**	**Operation**	**-Practical Example**
Binomial thinning operator Steutel and van Harn (1979) [10]	∘	$\alpha \circ X=\sum_{i=1}^{X}B_{i};\;\alpha \in \left[0,1\right)$ $B_{i}$ is the i.i.d Bernoulli random variables	• Daily count of visits to the web site of the “statistical calendar”. • Counts of utilization of the examination room of the emergency department of a children’s hospital. • Monthly counts of robbery.
Signed Binomial thinning operator Kim and Park (2008) [66]	⊙	$\alpha \;⊙X=\mathrm{sgn}\left(\alpha \right)\mathrm{sgn}\left(X\right)\sum_{i=1}^{\left|X\right|}W_{i};\;\alpha \geq 0$ $W_{i}$ is the i.i.d. Bernoulli random variables. sgn(x)=1ifx≥0, sgn(x)=−1ifx<0	• Monthly numbers of new patients diagnosed with AIDS
Signed generalized power series thinning operator Zhang et al. (2010) [67]	⊛	$\alpha ⊛X=sgn\left(\alpha \right)sgn\left(X\right)\sum_{i=1}^{\left|X\right|}W_{i};\;\alpha \geq 0$ $\left\{W_{i}\right\}$ is the i.i.d. generalized power series distribution.	• Count of drugs reported in the police car beat
Negative Binomial thinning operator Ristic et al., (2009) [15]	*	$\alpha *X=\sum_{i=1}^{X}G_{i};\;\alpha \in \left[0,1\right)$ $\;G_{i}$ is the i.i.d Geometric $\left(\frac{\alpha }{1+\alpha }\right)$ random variables	• Count of sex offences
Generalized Binomial thinning operator Ristic et al., (2013) [17]	°*θ*	$\alpha \circ_{\theta }X=\sum_{i=1}^{X}U_{i};\;\alpha \in \left[0,1\right),\;\theta \in \left[0,1\right]$ Here $U_{i}=\left(1-V_{i}\right)W_{i}+V_{i}Z$ where $\left\{V_{i},i\in N\right\}$ is the sequence of i.i.d. random variables with Bernoulli (θ) distribution, {W,i∈N} is the sequence of i.i.d. random variables with Bernoulli (α) distribution and Z is a random variable with Bernoulli (α) distribution.	• Monthly counts of vagrancy offences
Generalized Poisson thinning operator Kai Yang et al., (2019) [39]	⊛	$\left(\lambda ,k\right)⊛X=\sum_{i=1}^{X}C_{i}$ Here λ>0, max $(-1,\frac{-\lambda }{m})<k<1$ $\text{where}\;C_{i}$ is the i.i.d Generalized Poisson random variables	• Monthly claims count. • Monthly family violence counts.
GSC thinning operator Kang et al. (2020) [31]	♢	$\alpha ♢X=\sum_{i=1}^{X}W_{i};\;\alpha <1,\;\alpha \neq 0$ where $\left\{W_{i}\right\}$ is the sequence of i.i.d. GSC(α,exp{−|α|})	• Monthly count of criminal mischief. • Number of different IP addresses.
Generalized Negative Binomial thinning operator Shamma et al. (2020) [60]	${*}_{\theta }$	$\alpha {*}_{\theta }X=\sum_{i=1}^{X}U_{i};\;\;0\leq \alpha \leq \theta \leq 1$ Here $U_{i}=V_{i}Z$ where $\left\{V_{i},i\in N\right\}$ be a sequence of i.i.d. random variables with geometric $\left(\frac{\theta }{1+\theta }\right)$ distribution and Z is a Bernoulli $\left(\frac{\alpha }{\theta }\right)$ random variable (0≤α≤θ≤1).	• Weekly counts of Hantavirus disease.
Extended Binomial thinning operator Liu and Zhu (2021) [32]	⊚	$\left(m,\alpha \right)⊚X=\sum_{i=1}^{X}U_{i}\left(m,\;\alpha \right)$ [0≤α≤1,m≥2)] where {$U_{i}\left(m,\;\alpha \right)\}$ be a sequence of i.i.d. random variables with common distribution EB(m,1,α).	• Monthly tallies of crime data. • Stock data in New York Stock Exchange. • Crimes of family violence
GNTA thinning operator Amiri et al. (2024) [65]	${⊛}_{\theta }$	$\alpha {⊛}_{\theta }X=\sum_{i=1}^{X}U_{i};\;\alpha \in \left[0,1\right),\;\alpha \leq \theta e^{\theta }$ where $U_{i}$ is the dependent zero-inflated Bell distribution (ZIBe) random variable with parameters $\left(1-\frac{\alpha }{\theta e^{\theta }},\theta \right)$	• Monthly counts of drug calls. • Daily counts of COVID-19. • Weekly counts of Tularemia disease

The Table 2 summarizes studies from 2010 to 2024, highlighting the models used, estimation methods applied, performance measures evaluated for both simulation and empirical study, and the characteristics of count data addressed.

**Table 2:** Summary of INAR (1) models, estimation methods, performance measures and characteristics (2010–2024).

**Authors**	**Models used**	**Estimation Methods**	**Performance Measures**	**Characteristics of count data**
Mansour Aghababaei Jazi, Geoff Jones, Chin-Diew Lai (2012) [16]	GINAR (1), INAR (1)	CML, AFML	MSE, AIC	Overdispersion
Mansour Aghababaei Jazi, Geoff Jones, Chin-Diew Lai (2012) [47]	INAR (1), ZIP-INAR (1)	CML, AFML	MSE, AIC	Zero-inflation
Fukang Zhu (2012) [48]	Poisson INARCH (2), ZIP-INGARCH (2), NB1-INARCH (2), NB2-INARCH (2), ZINB1-INARCH (2), ZINB2-INARCH (2)	Expectation Maximization algorithm	MADE, AIC, BIC	Zero-inflation, overdispersion
Christian H. Weiß (2013) [27]	PINAR (1), GP-INAR (1), GP-INARCH, Good INAR (1), Power Law INAR (1)	MLE	AIC, BIC	Underdispersion
Sebastian Schweer, Christian H. Weiß (2014) [18]	Poi-INAR (1), INARCH (1), NB-INAR (1), Poi_2_-INAR (1), Poi_3_-INAR (1)	CML	AIC, BIC	Overdispersion
Ming Yang, Joseph E Cavanaugh, Gideon KD Zamba (2015) [49]	Dynamic models: Poisson + AR (1), NB + AR (1), ZIP + AR (1), ZINB + AR (1)	Monte-Carlo Expectation Maximization algorithm	AIC	Overdispersion, Zero-inflation
Miroslav M. Ristić, Aleksandar S. Nastić and Ana V. Miletić Ilić (2013) [17]	PINAR (1), NG-INAR (1), Mixed INAR (1), Mixed αβ INAR (1), NB-INAR (1), NB-INAR (1) I1, NB-INAR (1) I2, NB-INAR (1) I3, NBIINAR (1), NBRCINAR (1), DCGINAR (1), GPQINAR (1)	MLE, CLS	AIC, BIC, RMSE	Overdispersion
Marcelo Bourguignon (2015) [19]	PG-INAR (1), NG-INAR (1), INARCH (1), GINAR (1)	Two step CLS	MSE, RMSE, Absolute mean	Overdispersion
Marcelo Bourguignon, Klaus L. P. Vasconcellos (2015) [29]	PINAR (1), GINAR (1), NB-INAR (1), NG-INAR (1), Logarithmic INAR (1), Truncated INAR (1), ZTPINAR (1)	YW, CLS, CML	Bias, MSE, AIC, RMSE, Absolute mean	Overdispersion, underdispersion
Raju Maiti, Atanu Biswas, Samarjit Das (2015) [50]	ZIP-INAR (1), PINAR (1)	YW, CLS, MLE	PRMSE, PMAD, PTP, AIC	Zero-inflation
Wagner Barreto Souza (2015) [51]	ZMG-INAR (1), NG-INAR (1)	CLS	Standard Error	Zero-inflation, zero-deflation, overdispersion, underdispersion
Marcelo Bourguignon, Christian H. Weiß (2017) [36]	BerG-INAR (1) ^BiNB^, BerG-INAR (1) ^NB^	MLE	Bias, MSE, AIC	Equidispersion, underdispersion, overdispersion
Hanwool Kim & Sangyeol Lee (2017) [37]	PINAR (1), GINAR (1), NB-INAR (1), NBIINAR (1), NBRCINAR (1), GPQINAR (1), ZIP-INAR (1), NG-INAR (1), DCGINAR (1), KF-INAR (1),	CML, MLE	MSE, AIC, BIC, RMSE	Equidispersion, underdispersion, overdispersion
Marcelo Bourguignon (2018) [52]	ZIP-INAR (1), GINAR (1), ZMG-INAR (1)	CML, CLS, YW	MSE, AIC, BIC	Zero-inflation, zero-deflation, equidispersion, overdispersion, underdispersion
Marcelo Bourguignon, Josemar Rodrigues, Manoel Santos-Neto (2018) [38]	DP-INAR (1), GP-INAR (1), PINAR (1)	YW, CLS, CML	Bias, MSE, AIC, BIC	Equidispersion, underdispersion, overdispersion
M. Mohammadpour, Hassan S. Bakouch and M. Shirozhan (2018) [20]	PINAR (1), GINAR (1), NG-INAR (1), NBRCINAR (1), NBIINAR (1), GPQINAR (1), PL-INAR (1)	CLS, YW, MLE	AIC, BIC, RMSE, CAIC	Overdispersion
Tobias A. Moller,Christian H. Weiß, Hee-Young Kim, Andrei Sirchenko (2018) [53]	RZ-BAR (1), IZ-BAR (1), ZIB-AR (1), ZT^0^-BAR (1)	MLE	AIC, BIC	Zero-inflation
Marcelo Bourguignon,Patrick Borges, Fabio Fajardo Molinares (2018) [54]	PINAR (1), ZIP-INAR (1), NG-INAR (1), π-NGINAR (1)	MLE	MSE, AIC, BIC	Zero-inflation, zero-deflation
Hassan S. Bakouch, Mehrnaz Mohammadpour, Masumeh Shirozhan (2018) [55]	GINAR (1), NG-INAR (1), NBRCINAR (1), NBIINAR (1), ZMG-INAR (1), GINAR _RC_ (1), ZIG-INAR_RC_ (1)	MLE, CLS	RMSE, AIC, BIC, CAIC, HQIC	Zero-inflation
Raju Maiti, Atanu Biswas, Bibhas Chakraborty (2018) [7]	PINAR (1), GINAR (1), CP-INAR (1), ZIP-INAR (1), OMG-INAR (1)	YW, QMLE	MSE, PRMSE, PMAE, PTP, AIC	large number of zeros and ones
Kai Yang,Yao Kang, Dehui Wang, Han Li,Yajing Diao (2019) [39]	PINAR (1), GINAR (1), NG-INAR (1), GP-INAR (1)	CLS, MQL	Bias, MSE, RMSE	Overdispersion, underdispersion
Xiaohong Qi, Qi Li, Fukang Zhu (2019) [58]	INAR (1), OINAR (1), ZIP-NAR (1), ZOIP-INAR (1)	MLE	MSE, AIC, BIC	Overdispersion, underdispersion, excess zeros and ones
Mohammed Alqawba, Norou Diawara, N. Rao Chaganty (2019) [59]	Poisson, NB, ZIP, ZINB, ZICMP	MLE	MADE, AIC	Zero-inflation
Iain L. MacDonald, Feroz Bhamani (2020) [30]	Poisson-HMM, CMP-HMM	Moment Based estimation	AIC, BIC	Overdispersion, underdispersion
Lianyong Qian,Qi Li, Fukang Zhu (2020) [21]	DP-INAR (1), GP-INAR (1), GPIG-INAR (1), OMG-INAR (1), ZIP-INAR (1), ZOIP-INAR (1)	CML	MADE, MSE, AIC, BIC	Heavy-tailedness, equidispersion, overdispersion, zero-inflation,
Yao Kang, Dehui Wang, Kai Yang, Yulin Zhang (2020) [31]	GSCINAR (1), POINAR (1), NGINAR (1), ZMGINAR (1)	CLS, WCLS, MQL	MSE, RMSE	Overdispersion, underdispersion
Zhengwei Liu, Fukang Zhu (2020) [32]	INAR (1), ETINAR (1), GSCINAR (1), EB-INAR (1)	Two-step CLS, CML	RMSE, MAE, MADE, AIC, FMAE, FMSE	Overdispersion, underdispersion
Nisreen Shamma, Mehrnaz Mohammadpour, Masoumeh Shirozhan (2020) [60]	P-INAR (1), G-INAR (1), NBIINAR (1), NBRCINAR (1), NG-INAR (1), GPQINAR (1), DCGINAR (1), GADCINAR (1), NDCINAR (1), GNBINAR (1), ρ-GINAR (1), ρ-NGINAR (1), π-NGINAR (1)	CML, modified CLS, YW	RMSE, AIC, BIC, CAIC, HQIC	Zero-inflation
Enai Taveira da Cunha, Marcelo Bourguignon, Klaus L. P. Vasconcellos (2021) [60]	Borel INAR (1), TP-INAR (1), ZTP-INAR (1)	YW, CLS, CML	MSE, AIC, RMSE	Equidispersion, underdispersion, overdispersion
Saleh Ibrahim Musa, N. O. Nweze (2021) [22]	ACP, PAR	Not used	AIC, HQIC	Overdispersion
M. Sharafi, Z. Sajjadnia, A. Zamani (2023) [61]	GINAR (1), ZIP-INAR (1), ZMG-INAR (1), ZMPL-INAR (1)	YW, CLS, CML	Bias, MSE, AIC, BIC, HQIC, SMAPE	Zero-inflation, zero-deflation
Marinho G. Andrade, Katiane S. Conceição, Nalini Ravishanker (2023) [4]	deflated ZDP-GARMA (1,1), ZIP-GARMA (2,0), inflated ZINB-GARMA (1,1), ZDNB-GARMA (2,0), ZMNB-GARMA (3,0), ZMPS-GARMA	Bayesian methods via Hamiltonian Monte Carlo simulation algorithm.	RMSE, MAAPE, MAB, MAE, MAEM	Zero-inflation, zero-deflation
Eisa Mahmoudi, Ameneh Rostami (2023) [62]	ZMG-INMA (1), GINMA (1)	CML	Bias, MSE, RMSE	Zero-inflation, zero-deflation, equidispersion, overdispersion, underdipsersion
Lianyong Qian, Fukang Zhu (2023) [33]	ZIP-INAR (1), GPIG-INAR (1), COMP-INGARCH (1,1), GP-INGARCH (1,1) GCOMP-INGARCH (1,1), PINGARCH (1,1), NB1-INGARCH (1,1), NB2-INGARCH (1,1)	CML	MADE, MSE, AIC, BIC	Overdispersion, underdispersion, zero-inflation, heavy-tailedness
Yao Kang, Danshu Sheng, Feilong Lu (2024) [34]	PINAR (1), GINAR (1), GP-INAR (1), ZIP-INAR (1), CP-INAR (1), CPRCINAR (1), ZTP-INAR (1), GPIG-INAR (1), PBE_2_-INAR (1), Borel INAR (1), ZDGG-INAR (1)	YW, CML, CLS, Bayesian method	MSE, MADE, AIC, BIC, RMSE	Overdispersion, underdispersion, zero inflation, zero-deflation, heavy-tailedness, zero-truncation
N. Balakrishna, P. Muhammed Anvar, Bovas Abraham (2024) [63]	ZIPSCD-GAR (1), ZINBSCD-GAR (1), ZDPSCD-GAR (1), ZDNBSCD-GAR (1),	Estimating Functions based estimation	MSE, Probability Integral Transform	Zero-inflation, zero-deflation
Abderrahmen Manaa, Roufaida Souakri (2024) [64]	INAR (1), GP-INAR (1), ZIP-INAR (1), PZIP-INAR_7_(1), PINAR_7_(1)	CML, CLS, YW	AIC, BIC, RMSE	Zero-inflation
J. Amiri, R. Farnoosh & M.H. Behzadi (2024) [65]	PINAR (1), GINAR (1), NGINAR (1), NBIINAR (1), NBRCINAR (1), GPQINAR (1), NDCINAR (1), DCGINAR (1), GADCINAR (1), GNBINAR (1), GNBTGINAR (1), ρ-GINAR (1), ρ-NGINAR (1), π-NGINAR (1)	CML, YW, modified CLS	AIC, BIC, HQIC, CAIC, RMSE,	Zero-inflation

Across the existing literature, a consistent pattern emerges in favor of CML estimation when compared with CLS, YW and related alternatives. Many studies show that CLS and YW estimators tend to exhibit very similar numerical behavior, particularly in terms of MSE, but are generally outperformed by CML, especially under characteristics such as zero-inflation, zero-deflation, and varying dispersion levels ([29,38,61]). The superiority of CML is largely attributed to its use of the full distributional information, leading to smaller bias, MSE, and standard errors, as well as faster convergence toward true parameter values ([52,60,64]). Although CLS is often highlighted for its closed-form structure and computational simplicity, its performance is typically close to that of CML. However, it remains slightly inferior, particularly in large samples where all estimators are asymptotically unbiased [40]. While recent study suggests that both CML and Bayesian approach yield satisfactory results, with their performance becoming nearly identical in large-sample settings [34]. Overall, among estimation methods, with CML providing the most reliable and efficient performance, followed by CLS, whereas YW-based estimators generally display the weakest empirical properties.

## Software resources and practical implementation

Several R packages have been developed to support the analysis of count time series data. Table 3 summarizes commonly used R packages and their primary functionalities, offering readers practical guidance for selecting appropriate R package for count time series analysis.

**Table 3:** R packages for count time series analysis.

**R package**	**Description**
fableCount	INGARCH and GLARMA models for count time series
forecast	Forecasting functions for time series models and linear models
Gcmr	Gaussian copula marginal regression
INLA	Modeling count time series
PNAR	Network time series models for count data
spINAR	(Semi-)parametric estimation and bootstrapping of INAR models
Tbats	Advanced forecasting for count data
tscount	Analysis of count time series following generalized linear models
tsinteger	Time series analysis of count data
tsglm	Generalized linear models for time series
ZIM	Zero-inflated models for count time series with excess zeros
ZINARp	Zero-inflated INAR models

Practical implementation resources for INAR (1) models are available in the textbook *An Introduction to Discrete-Valued Time Series* by [68]. This reference provides R code and datasets for classical INAR (1) models, covering both parameter estimation and statistical inference. To facilitate reproducibility and allow readers to replicate, modify, and extend the computations, all R codes are provided through the companion website (www.wiley.com/go/weiss/discrete-valuedtimeseries) as a password-protected ZIP archive (DiscrValTS17). The corresponding datasets are also freely available from the same website. In addition, the GitHub repository *GitHub - projecttsinteger/tsintegerpackage: R package for analysis of time series of counts* provides code for estimation methods, hypothesis testing, and simulation studies, along with selected datasets for Poisson-INAR(1) and NG-INAR(1) models.

Although several R packages are available for analyzing count time series data, most of them support classical INAR models or GLM-based frameworks. A major computational challenge arises for recently developed INAR (1) models, such as ZDGG-INAR (1), GPIG-INAR (1), GP-INAR (1), DP-INAR (1), and others, which are capable of handling multiple characteristics of count data. These advanced models are often not implemented in existing software packages. As a result, parameter estimation and inference frequently require manual coding of likelihood functions and estimation algorithms. This limits the ease of implementation and may hinder the practical application of newly proposed INAR (1) models, particularly for applied researchers.

## Future research directions

1. Development of hybrid models by integrating INAR model with neural networks and deep learning techniques, to improve predictive performance and capture nonlinear dependencies in high-dimensional count data.
2. Investigate higher-order INAR models (order >1) across different types of count data to assess their performance in capturing complex temporal dependencies.
3. Extend INAR models to handle multivariate and high-dimensional count time series to address the complexity of real-world data.
4. Development of R or Python packages could facilitate practical implementation and reproducibility.
5. Modify or generalize the innovation distribution to accommodate overdispersion, excess zeros, or heavy tails, enabling a single INAR model to capture diverse features of count data.
6. Improve estimation methods, particularly through the advancement of Bayesian approaches, to enhance model flexibility and inference accuracy.
7. Introduce novel thinning operators to better capture different forms of dependence and distributional properties in count data.

## Conclusion

This review provides valuable insights into recent advancements in INAR models for count time series data, covering developments from 2010 to 2024. As a significant contribution, this article emphasizes the impact of key characteristics including equidispersion, overdispersion, underdispersion, and zero-inflation/deflation on model performance and parameter estimation. By addressing a broad range of characteristics within a single article, the review guides researchers in selecting appropriate models and estimation methods that align with specific data characteristics, enhancing accuracy in estimation and forecasting. Through an innovative approach, this review examines the strengths and weaknesses of models suited to both single and multiple data characteristics, including combinations of dispersion and inflation, providing a strong foundation for identifying research gaps and opportunities for improvement.

Additionally, the review highlights recent methodological improvements that offer robust solutions for complex data scenarios. Leveraging these advancements enables researchers to better manage the challenges posed by diverse count data characteristics, leading to more accurate forecasts, reliable inferences, and informed decision-making.

## Ethics statements

Not applicable.

## Supplementary material *and/or* additional information [optional]

None.

## CRediT authorship contribution statement

**Vinitha Serrao:** Methodology, Writing – original draft, Writing – review & editing. **Satyanarayana Poojari:** Conceptualization, Supervision, Writing – review & editing. **Asha Kamath:** Supervision, Writing – review & editing.

## Declaration of competing interest

The authors declare that they have no known competing financial interests or personal relationships that could have appeared to influence the work reported in this paper.
